# Prediction of IGBT Gate Oxide Layer’s Performance Degradation Based on MultiScaleFormer Network

**DOI:** 10.3390/mi15080985

**Published:** 2024-07-30

**Authors:** Shilie He, Meiling Yu, Yiqiang Chen, Zhenwei Zhou, Lubin Yu, Chao Zhang, Yuanhua Ni

**Affiliations:** 1 School of Aeronautics, Northwestern Polytechnical University, Xi’an 710072, China; heshilie@126.com (S.H.);; 2 China Electronic Product Reliability and Environmental Testing Institute, Guangzhou 511370, China; 3 Key Laboratory of Science and Technology on Reliability Physics and Application of Electronic Component, Guangzhou 511370, China; 4 School of Artificial Intelligence, Nankai University, Tianjin 300350, China; yumeiling0512@126.com (M.Y.);

**Keywords:** IGBT, gate oxide layer degradation, feature fusion, performance prediction, MultiScaleFormer network

## Abstract

Insulated gate bipolar transistors (IGBTs) are widely used in power electronic devices, and their health prediction problems have attracted much attention in the field of power electronic equipment health management. The performance degradation of IGBT gate oxide is one of the most important failure modes. In order to analyze this failure mechanism and the ease of implementation of a monitoring circuit, the gate leakage current of IGBTs was selected as the fault precursor parameter for the degradation of their gate oxide performance, and feature selection and fusion were carried out by using time domain characteristic analysis, grayscale correlation, Mahalanobis distance, Kalman filter, and other methods. Thus, a health indicator was obtained to characterize the degradation of IGBT performance, which was used to indicate the degree of aging of the IGBT gate oxide layer. In this paper, we propose an improved degradation prediction model called MultiScaleFormer, inspired by advanced design ideas of the iTransformer network architecture, combined with the health parameters of IGBTs to construct a degradation prediction model for the IGBT gate oxide layer. MultiScaleFormer showed the highest fitting accuracy compared with the Long Short-Term Memory (LSTM), Convolutional Neural Network (CNN), Support Vector Regression (SVR), Gaussian Process Regression (GPR), CNN-LSTM, and Transformer models in our experiment. The mean absolute error (MAE) of the MultiScaleFormer prediction was as low as 0.0087. Extraction of the health indicator and the construction and verification of the degradation prediction model were carried out on the dataset released by the NASA-Ames Laboratory. These results demonstrate the feasibility of the gate leakage current as a fault precursor parameter for IGBT gate oxide failure, and the feasibility and accuracy of the MultiScaleFormer prediction model for IGBT performance degradation.

## 1. Introduction

The function of insulated gate bipolar transistors (IGBTs) is to realize energy conversion output, and they are key power semiconductor components in power conversion equipment. IGBTs combine the advantages of MOSFET and bipolar transistors, and have the advantages of a high input impedance, fast switching speed, low switching loss, high current density, saturation voltage reduction, and strong current control ability. In recent years, IGBTs have been widely used in military and economic fields such as aerospace, maritime transportation, rail transit, and new energy power generation, and are expected to maintain a stable growth trend in the future, with an irreplaceable status and role [1,2,3]. Due to the rapid development of power semiconductor manufacturing technology, the size of IGBT modules is becoming smaller, but their external load conditions are growing heavier. With their high failure rate, the contradiction between improving the reliability of power conversion devices and the fatigue failure of IGBTs is becoming increasingly prominent. As a key aspect, the aging failure of a power conversion device will affect the normal operation of the whole system, and even cause huge economic losses or catastrophic consequences in serious cases. How to predict the failure of IGBTs in advance is the key, and a reliable fault prediction system can predict the degradation trend of IGBTs in their whole life cycle and provide alarm information to assist the system to sense in advance and take maintenance measures to avoid serious consequences.

Both reliability-based life prediction and data-driven methods based on fault precursor parameters have been applied in IGBT fault prediction [4,5]. Reliability-based life prediction methods [6,7,8] include physical analysis models and experimental statistical models. Experimental statistical models rely on equivalent conversion from actual working conditions to aging test conditions, and their accuracy is generally low. Physical analysis models are based on the IGBT failure mechanism, predicting faults through multiple physical field stress distribution, material parameters, damage, and other parameters. It is necessary to establish accurate mechanism models and parameters, and there are also great difficulties and uncertainties in the engineering application of time. Data-driven methods based on fault precursor parameters refer to predicting faults based on the trends of changes in variables that can measure the IGBT performance and state, as well as fault symptoms. At present, in engineering applications and research on fault prediction, the most used method needs to select the fault-sensitive parameters of IGBTs or the performance and state variables directly or indirectly affected by the fault for monitoring. The parameters that characterize IGBT degradation and even failure include dynamic parameters and static parameters, where static parameters include the gate threshold voltage (V_GE.th_) and gate-emitter peak voltage (V_GES_), etc., and dynamic parameters include the gate-emitter leakage current (I_GES_), turn-off time (T_OFF_), collector-emitter saturation voltage (V_CE.sat_), thermal resistance, etc. Without the need to grasp the accurate mechanism of IGBT internal faults, the historical data collected by sensors can be used to characterize the degree of chip and bond aging failure of IGBTs, so as to carry out more accurate predictions. Data-driven methods include statistics-based failure prediction methods and artificial-intelligence-type algorithms.

In recent years, with the rapid development of artificial intelligence algorithms and computer computing power, many new and complex methods such as neural networks (NNs) [9,10,11], support vector machines (SVMs) [12], and deep learning (DL) have been used to construct prediction methods. The current research mainly extracts characteristic parameters from collector-emitter voltage data and predicts the degradation trend of IGBTs, and the research on IGBT health assessment and prediction mainly focuses on failure modes such as the latch-up effect and bond line failure of IGBT chips. A series of modeling analyses were carried out mainly for the collector-emitter voltage. Ge et al. used the peak-to-peak voltage of the transient collector-emitter voltage as an indicator of the IGBT performance degradation and published a DeepAR prediction model in [13] to predict the lifetime of IGBTs. Lu and Christou also extracted performance degradation metrics from collector-emitter voltages and used a long short-term memory (LSTM) prediction model to predict the performance degradation trend of IGBTs, where the mean absolute error was predicted to be as low as 0.0322 [14]. Zhang et al. [15,16] extracted parameters such as the saturation voltage drop and tailing current for fusion, and introduced comprehensive health indicators to obtain the results of an IGBT health status assessment. Liu et al. [17] introduced the CNN1D-LSTM hybrid model to construct an IGBT lifetime prediction model for collector-emitter voltage, which characterizes the solder layer failure and latch-up failure modes of IGBTs. Wang et al. presented the CNN1D-LSTM model to predict the gate leakage current of IGBTs and achieved leading accuracy results [18]. 

Gate oxide degradation is one of the main failure mechanisms of IGBTs. This failure mechanism can lead to IGBT gate turn-on-off faults, which can be characterized at the signal level by the I_GES_ and V_GE.th_ parameters of the IGBT. Due to the fact that the collection of IGBT collector-emitter voltage involves high-voltage testing in engineering practice, the measurement circuit is relatively complex, and the on and off times are in the microsecond range, requiring the use of high-speed data acquisition devices to capture them. The I_GES_ acquisition circuit for monitoring IGBT is relatively simple and small in size, which provides engineering application advantages. Moreover, this parameter can directly reflect the gate control performance of IGBTs. Therefore, this article selects I_GES_ as the precursor parameter of IGBT gate oxide degradation for feature extraction and degradation trend modeling. This article uses the dataset released by NASA’s Ames Laboratory, which is always used for competions of the aging prediction in public. Firstly, a time domain analysis of I_GES_ is carried out and combined with the grey correlation degree, Mahalanobis distance, and Kalman filtering method. Multi-dimensional features are fused into a unique health indicator to characterize the degree of degradation of the IGBT gate oxide. Secondly, this article introduces advanced network models such as transformers to achieve the real-time prediction of performance degradation, and based on this, we propose a model with a better performance through network optimization, which we call MultiScaleFormer. It shows an excellent fitting ability in predicting IGBT degradation. Finally, this article verifies the effectiveness of the MultiScaleFormer network and discusses the prediction errors using several different prediction models.

Compared with the previous work, this paper has several innovations, which are as follows: a new method was presented with a cross-attention mechanism to simultaneously focus on information from different time scales for the prediction of the IGBT gate-emitter leakage current. The architecture of the model was optimized to improve its ability to handle complex time series data. A better prediction accuracy was achieved by capturing both multidimensional and temporal correlations.

## 2. Analysis of IGBT Failure Modes and Their Precursor Parameters

The degradation of IGBT health state is related to the stress of its working profile, showing a gradual degradation process. Changes in IGBT precursor parameters are monitored in real time through condition-monitoring technology, and potential failure sharing is detected in advance when the IGBT health state is close to failure. The key is to clarify the failure mechanism of the IGBT and the correspondence between related sensitive parameters. 

### 2.1. Failure Mode of IGBTs in Application

The failure modes of IGBTs include random failure and fatigue failure. Random failure is a transient failure type, mainly caused by over-electric stress or electrostatic discharge (ESD), etc., while fatigue failure belongs to degenerative failure, which is subdivided into chip internal fatigue failure and device package fatigue failure. The internal fatigue failure modes of the chip include ion diffusion due to interface fatigue, increases in charge surface density, increases in leakage current at the emission interface, and decreases in carrier mobility due to the fatigue of silicon materials. Package-related failures are mainly due to the different thermal expansion coefficients of the different materials such as silicon and metal in IGBT packages, and the structure of the connectors is constantly affected under the action of thermal stress, which even leads to failure phenomena such as bond wire detachment, solder layer aging, and aluminum metal reconstruction.

### 2.2. Selection of Precursor Parameters for Gate Oxide Layer Degradation

Precursor parameters can directly or indirectly reflect the health statuses of IGBTs [7,8,9,10], and their types are described in Section 1 of this article.

Junction temperature and thermal resistance are mainly used to characterize package-related failure modes such as the degradation of IGBT solder layers, bond wires, etc., in order to not interfere with the normal operation of IGBTs. Non-invasive measurement methods are often required to calculate the junction temperature and power loss by building accurate power consumption and heat transfer models.

The turn-on and turn-off times are mainly used to characterize the fatigue degradation of the chip, and an increase in the threshold voltage caused by a change in the interface state of the IGBT is reflected in increases in the turn-on and turn-off time.

The saturation voltage drop can be used to characterize and warn about the fatigue degrees of key wires, solder joints, and other parts. The continuous impact of thermoelectric stress will lead to an increase in the equivalent resistance and the loss of the energizing situation will increase, thereby causing an increase in the collector-emitter saturation voltage.

The part of the shutdown current IC in the IGBT slow drop phase is called the tail current, and the shutdown current duration can be used as a performance parameter to characterize the IGBT latch-up effect, so the tailing current can also be used as an electrical parameter to characterize the IGBT latch-up effect.

The gate leakage current (I_GES_) and gate-emitter threshold voltages (V_GE.th_) can be used to characterize the gate oxide degradation in IGBT devices [11,12]. With a change in the aging process of the device, the degradation of the IGBT performance will affect the internal structure of the gate oxide layer of the device, and a change in the oxide layer will also lead to a change in the gate capacitance parameters. Although V_GE.th_ can be monitored under laboratory conditions by certain techniques, V_GE.th_ is a static parameter that needs to be measured by a step-by-step approximation test method, so it is not suitable for implementation in the field of practical application. In contrast, the sensing measurement and conditioning circuit of I_GES_ can be realized as the main precursor parameters to evaluate the degradation of the gate oxide performance of IGBT devices in engineering.

Changes in the gate leakage current characteristics of IGBTs are mainly due to the degradation of gate oxide performance, the inherent pinhole defect of the gate dioxide film insulation layer, or the later fatigue aging. This will lead to ion diffusion at the gate Al-SiO2 interface and increase the surface charge density of Si-SiO2 in IGBTs. This is due to the presence of an electron, hole, and neutral traps in the gate oxide layer, which trap carriers and accumulate positive or negative charges when an oxide tunnel has an electric current passing through its well. The accumulated ionic charge, interface trap charge, oxide trap charge, etc., will enhance the local electric field of the oxide layer, further increase the leakage current, and finally, the gate oxide layer will break down, resulting in gate control failure. In summary, the precursor parameters of IGBT gate oxide attenuation failure are the gate-emitter threshold voltage and gate leakage current. Since it is easy to achieve the on-line detection required to collect the gate leakage current in engineering, the gate leakage current is used as a precursor parameter to evaluate and predict IGBT degradation.

In summary, I_GES_ is suitable for use as the precursor parameter of the performance degradation failure of the IGBT gate oxide layer. The performance degradation indicator of IGBTs is obtained by time domain analysis, feature selection, fusion, and noise reduction.

## 3. Indicator of IGBT Gate Oxide Layer Performance Degradation

According to the degradation mechanism of the IGBT chip interface material, for the performance degradation of the IGBT gate oxide layer, the performance degradation i indicator of the gate leakage current can intuitively characterize the aging degree of the IGBT gate oxide layer, and we will use this as the input for the prediction model, When the model prediction results show the failure threshold, this means that the IGBT is about to fail, and the IGBT needs to be replaced or other corresponding maintenance measures need to be taken.

In this article, we use the IGBT aging experimental data published by NASA, select the last 700 cycles at the end of the experiment for empirical analysis, and exclude the last 10 cycles after IGBT failure. A set of turn-on and turn-off actions is equivalent to a single cycle. When the IGBT gate is turned on, the current flowing through the gate is the operating current, and when the IGBT is turned off, the small current flowing through the gate is the leakage current.

**Figure 1:**
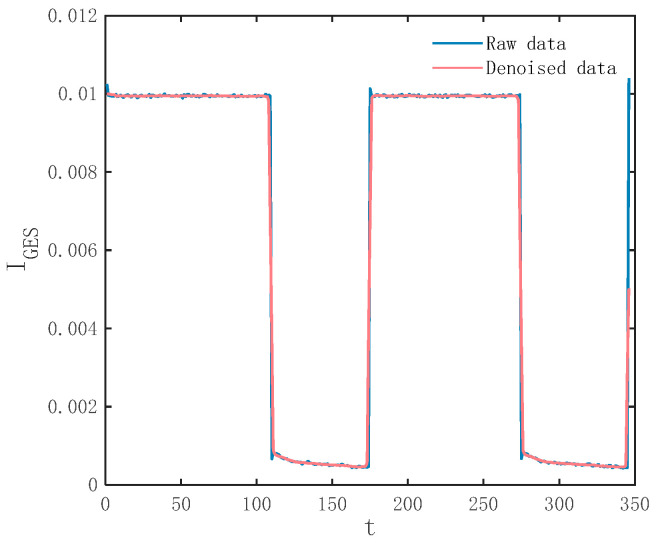
Signal pre-processing of I_GES_.

Before I_GES_ information acquisition, considering the effect of signal noise, we pre-process the original signal to improve the SN ratio by the NLM (non-local mean) filtering algorithm. This removes noise in a non-local mean by chunking the signal and calculating the similarity between each block and the others, with the advantage of removing noise while still preserving the edge and detail features of the signal. The signal of I_GES_ before and after filtering is shown as Figure 1.

Subsequently, time domain features, such as the maximum, minimum, variance, and skewness, are extracted through the time domain analysis of I_GES_, and then the IGBT performance degradation indicator MD is obtained by feature selection, fusion, and noise reduction. The subsequent performance prediction is carried out based on the degradation indicator MD. The reason for the selection of multiple features is that not all the extracted time domain features contain the degradation information of the IGBT gate oxide layer, and the similarity of the change trends of different features of the IGBT gate oxide degradation is characterized, so we have to sift. On the basis of clarifying the time domain feature group associated with the degradation characteristics of I_GES_ signals, it is necessary to form a unified indicator to characterize the performance degradation of IGBTs, and at the same time, due to the large amount of noise introduced in the data acquisition process, the trend fitting of the signal is adversely affected and the data need to be reprocessed and filtered. In this article, a feature matrix is constructed based on the Mahalanobis distance (MD), and the Kalman filter is used to denoise the noise. Reducing the signal noise in the acquired data has a higher accuracy and better interpretability in the field of signal processing, and the greater the Mahalanobis distance (MD) after data processing, the greater the deviation between sample sets, which is consistent with the degradation process trend of IGBTs.

**Figure 2:**
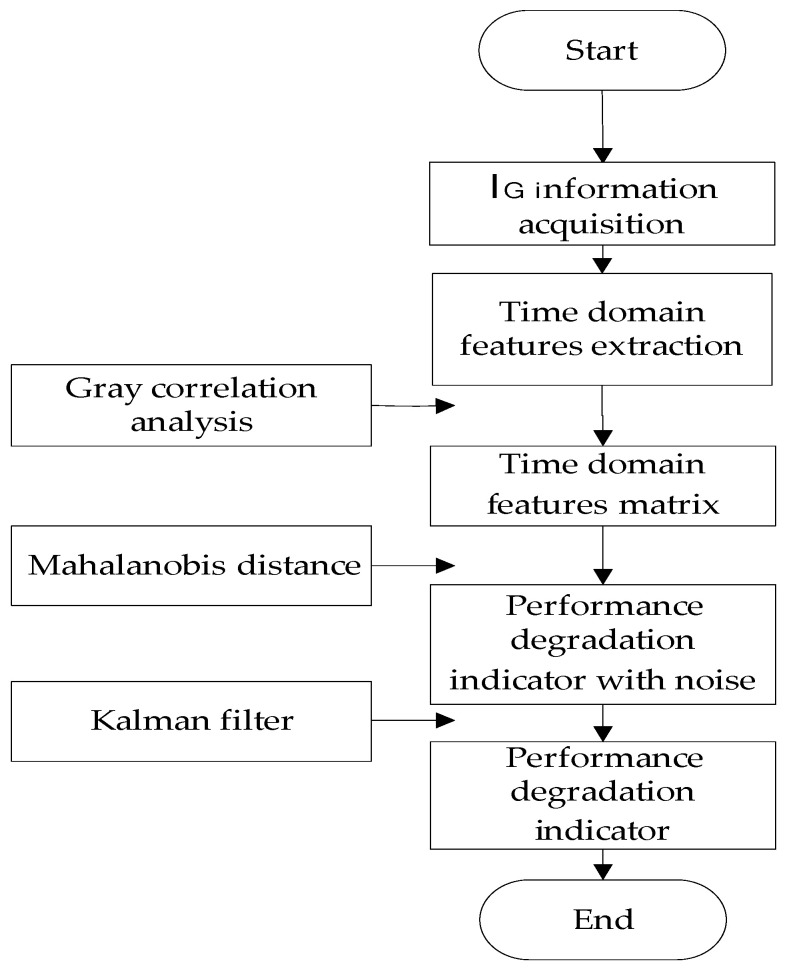
Construction of performance degradation indicator of IGBTs.

**Figure 3:**
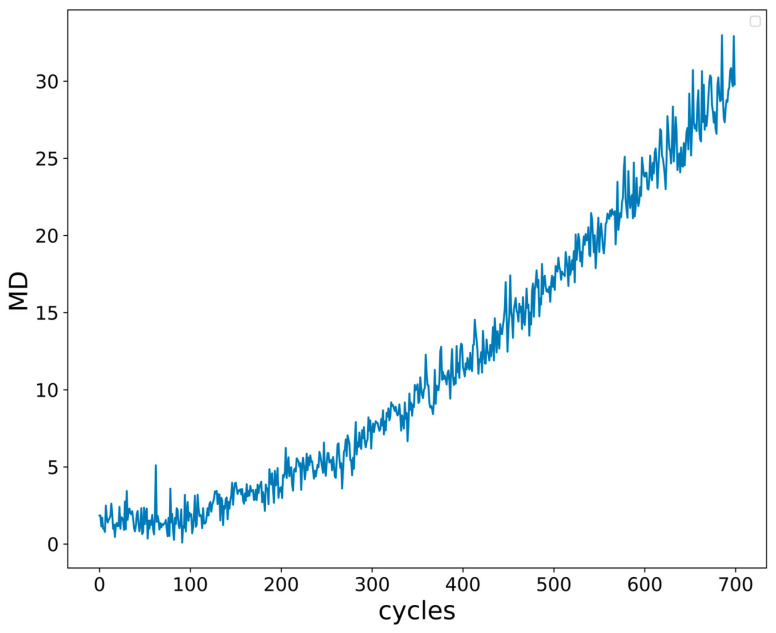
Mahalanobis distance value of I_GES_.

The construction flow chart of the performance degradation indicator of IGBTs is shown in Figure 2. The final indicator for the IGBT gate oxide degradation is shown in Figure 3.

## 4. Improved Transformer for Gate Oxide Performance Degradation Prediction

In the previous sections, we discussed the critical issue of IGBT performance degradation and conducted a time domain analysis of I_GES_ to predict the degradation of the IGBT gate oxide layer. By using methods such as grey correlation, Mahalanobis distance, and Kalman filtering, we extracted and fused multi-dimensional features to obtain a unique health indicator that characterizes the degree of this degradation. This section will discuss the improved Transformer-based prediction model for IGBT gate oxide layer performance degradation, using the previously obtained health indicator as the input for the prediction model.

A time series is a discrete representation of a stationary process with dynamics or system characteristics. The dataset used in this paper is NASA’s IGBT aging experiment data, which show aging experiments throughout the IGBT life cycle, using sensors to monitor the performance parameters in real time. The interval time of the data points that we used is changed from the interval time when the original data are collected by the sensor to the cycle time of the IGBT. During a cycle, the IGBT undergoes a set of turn-on and turn-off times. The effective length of the dataset is 700 consecutive cycles, which is close to covering the entire aging test time. On this basis, it means that it does not destroy the time series of the dataset, so the health indicator we obtained in the previous sections is a one-dimensional feature matrix of the input prediction model and can still be regarded as a time series.

The Transformer is an excellent model which is used for time series prediction. Since its introduction in 2017, the Transformer model [19] has become increasingly popular for time series analysis due to its unique architecture and capabilities. The core application of the Transformer in time series analysis lies in its self-attention mechanism, which effectively captures the long-term dependencies in time series data. However, due to the strict and fixed nature of time correlations, the flexibility of the attention mechanism can lead to disruptive effects when dealing with strictly ordered data. To address this, we propose an improved algorithm called MultiScaleFormer, inspired by iTransformer [20]. This algorithm processes input channels to capture variable dimensional correlations using attention mechanisms and temporal correlations with feedforward neural networks. To effectively integrate multi-scale information, we adopt a cross-attention mechanism [21] that allows for information exchange between different time steps, layers, or scales, providing a greater flexibility and adaptability, thus ensuring prediction accuracy.

### 4.1. Transformer Model

For predicting IGBT performance degradation, traditional models like Recurrent Neural Networks (RNNs) and their improved versions, such as Long Short-Term Memory (LSTM) and Gated Recurrent Units (GRU), process data sequentially. Each time step depends on the previous one, leading to two significant issues: (i). Slow Training and Inference: they cannot fully leverage parallel processing capabilities, resulting in longer training and inference times, which pose challenges for real-time IGBT monitoring. (ii). Poor Scalability: handling very long sequences or large datasets incurs high computational costs and time. Additionally, gradient vanishing issues make it difficult for the model to learn long-term dependencies, which are critical in capturing the gradual degradation of IGBTs.

To overcome these challenges, we adopt the Transformer model, a significant advancement in time series analysis due to its innovative self-attention mechanism and parallelizable architecture. Unlike RNNs, the Transformer model efficiently handles long-range dependencies and supports parallel processing, thereby significantly enhancing training efficiency.

**Figure 4:**
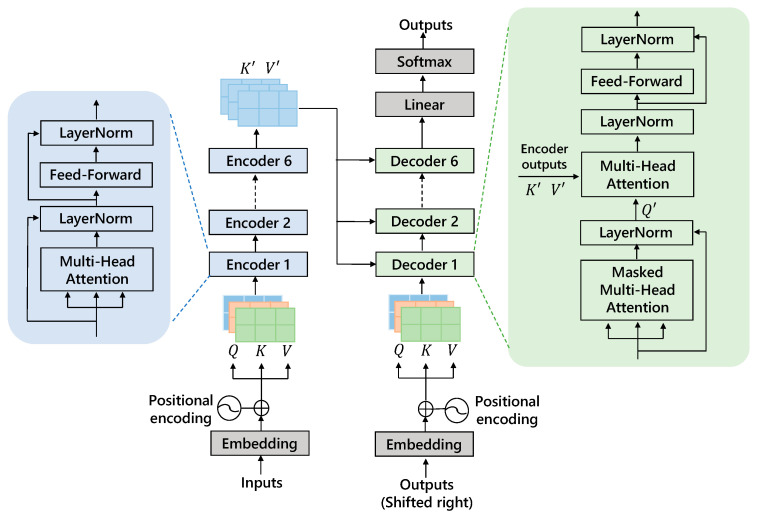
Overall structure of Transformer.

The Transformer model architecture, as shown in Figure 4, mainly consists of four parts: an input module, encoder module, decoder module, and output module, with the core being the encoder-decoder part. The encoder consists of six encoder blocks, and the decoder consists of six decoder blocks, with the encoder’s output serving as the decoder’s input. Each encoder and decoder contain two main sub-layers: a multi-head self-attention mechanism and a fully connected feed-forward network.

**Figure 5:**
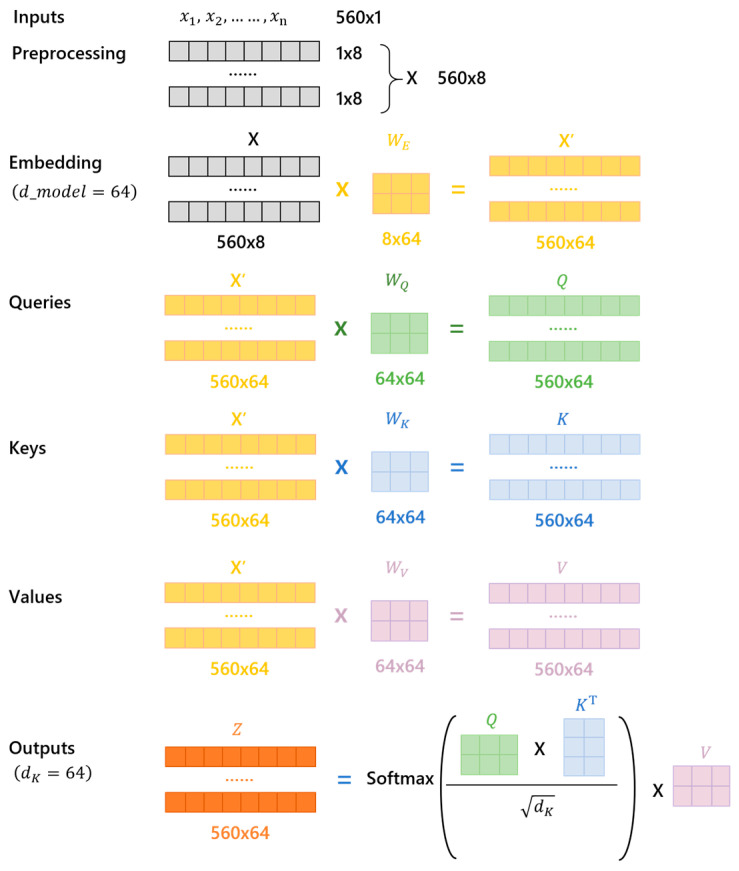
An example analysis of the self-attention mechanism in IGBT gate current Ppediction.

The self-attention mechanism within the Transformer model allows it to dynamically weigh the importance of different time steps in the sequence of IGBT gate current data. This mechanism enables the model to capture the complex dependencies and interactions between different time steps and features, leading to more precise predictions of the IGBT performance degradation. In self-attention, each word has three different vectors: a query vector (*Q*), key vector (*K*), and value vector (*V*). These vectors are obtained by multiplying the embedding vector X with three different weight matrices $W^{Q}$, $W^{K}$, and $W^{V}$. After passing through the self-attention module, a weighted feature vector *Z* is obtained, calculated as follows:

(1) $$Z=\mathrm{Attention}(Q,K,V)=\mathrm{softmax}(\frac{QK^{T}}{\sqrt{d_{k}}})V$$

where $d_{k}$ is the dimension of the keys and the softmax function is used to normalize the attention weights. Figure 5 briefly describes the process of using the Transformer for predicting the gate current, particularly the calculation of *Q*, *K*, and *V* in the self-attention mechanism and the derivation of the output vector *Z*.

The model input consists of gate current data from the training set, including 560 sequences. The input data undergo preprocessing to form a 560 × 8 matrix X. This matrix is then passed through an embedding layer, resulting in a 560 × 64 matrix X’. The embedding matrix X’ is used to generate the query (*Q*), key (*K*), and value (*V*) matrices by multiplying X with their respective weight matrices $W^{Q}$, $W^{K},$ and $W^{V}$, each resulting in a 560 × 64 matrix. The self-attention mechanism computes the dot product of *Q* and the transpose of *K*, scales them by the square root of the key dimensionality, and applies a softmax function to obtain attention scores. These scores are then used to weigh *V*, resulting in the final output matrix *Z* with the shape 560 × 64. This mechanism enables the model to effectively capture dependencies within the input sequences for subsequent prediction tasks.

To enhance the model’s ability to focus on different positions, the Transformer uses a multi-head attention mechanism, allowing the model to jointly attend to information from different representation subspaces. This is achieved by concatenating the outputs of h self-attention heads and projecting them back to the original space. The output of multi-head attention is divided into three steps: (i). The input data X are linearly projected into multiple subspaces, each corresponding to an independent self-attention head, with a total of h heads. (ii). Self-attention is calculated independently in each subspace, resulting in h weighted feature matrices $Z^{i}$. (iii). These h feature matrices are concatenated together to form a new feature matrix, which is then passed through a linear transformation to obtain the final output *Z*.

Layer normalization is a key component in the Transformer model. By normalizing the output of each layer to have a zero mean and unit variance, this improves the stability of model training. Unlike batch normalization, layer normalization does not depend on batch data, making it suitable for sequence modeling tasks. In the Transformer, layer normalization is typically applied after the self-attention mechanism and feed-forward network. Each layer’s output is normalized and then added to the input via residual connections, forming the final layer output. Assuming that the input is *x*, it can be expressed as:

(2) $$\mathrm{LayerNorm}(x)=\gamma \frac{x-\mu }{\sqrt{\sigma^{2}+\varepsilon }}+\beta$$

where μ and $\sigma^{2}$ are the mean and variance, ϵ is a small constant to prevent division by zero, and γ and β are trainable parameters for scaling and shifting.

Each encoder and decoder contain a fully connected feed-forward network (FFN) applied independently and identically to each position. These consist of two linear transformations with a ReLU activation function in between, formally expressed as follows:

(3) $$\mathrm{FFN}(x)=\max(0,xW_{1}+b_{1})W_{2}+b_{2}$$

where $W_{i}$ and $b_{i}$ represent the weights and biases of the *i*-th linear layer, respectively.

The Transformer model innovatively uses the self-attention mechanism and its parallelizable architecture to reduce computational complexity, achieving a better performance than RNNs in handling temporal data and sequence modeling. Despite these strengths, the standard Transformer model has limitations when applied to time series data, especially in capturing the strict temporal correlations inherent in IGBT degradation processes. To address these issues and further improve performance, we propose an enhanced model called MultiScaleFormer.

### 4.2. MultiScaleFormer Architecture

Building upon the foundation of the Transformer model, we introduce the MultiScaleFormer to overcome its limitations and enhance its applicability to time series data. The MultiScaleFormer is designed to better capture variable dimensional correlations and strict temporal dependencies, ensuring more accurate and reliable predictions. It is employed to construct a robust prediction model for IGBT performance degradation. The model input is a one-dimensional IGBT performance degradation indicator matrix $X\in {\mathbb{R}}^{T\times V}$, where T represents the time steps and V represents the variables. The output targets is an IGBT real-time degradation metric $Y\in {\mathbb{R}}^{N\times V}$ for N future time steps.

Most Transformer-based prediction models typically treat multiple variables at the same time as a single token. To better capture the correlations among variable dimensions, we treat each variable at different time steps as a separate token and use a multi-layer perceptron (MLP) for embedding. Specifically,

(4) $$\begin{array}{l} {h_{v}}^{\left(1\right)}=\sigma (W_{1}x_{v}+b_{1})\\ {h_{v}}^{\left(i\right)}=\sigma (W_{i}{h_{v}}^{\left(i-1\right)}+b_{i})\;i=2,\ldots ,L-1\\ \;e_{v}=W_{L}{h_{v}}^{\left(L-1\right)}+b_{L} \end{array}$$

where $x_{v}={\left[x_{v}(1),\ldots ,x_{v}(T)\right]}^{T}$ represents the time series data of the v-th variable, the MLP has *L* layers with weights and biases for each layer denoted by $W_{i}$ and $b_{i}$ respectively, ${h_{v}}^{\left(i\right)}$ is the output of the i-th layer, and $e_{v}$ represents the embedding vector of $x_{v}$. Additionally, the ReLu function is used as the activation function σ in this experiment.

Positional encoding adds positional information to the input data, enabling the model to distinguish inputs from different time steps, which is crucial for time series prediction. By understanding the relationships between the inputs at current and previous time steps, the model can better learn patterns and trends in the time series, improving its prediction performance. Positional encoding is particularly useful for understanding the relationships between distant time steps in long sequence data.

The input first passes through a time attention mechanism layer, independently encoding each sequence temporally and processing the encoded information through layer normalization and MLP. The fused data are then input into a dimension attention mechanism layer to align the encodings of different variables across all time steps, returning the final output and attention weights.

(5) $$\begin{array}{cc} \mathrm{Time}\_\mathrm{Enc},\mathrm{Attn} & =\mathrm{TimeAttention}(X)\\ \mathrm{Time}\_\mathrm{Emb} & ={\mathrm{Norm}}_{2}({\mathrm{MLP}}_{1}{(\mathrm{Norm}}_{1}(\mathrm{Time}\_\mathrm{Enc})))\\ \mathrm{Dim}\_\mathrm{Enc} & =\mathrm{DimAttention}(\mathrm{Time}\_\mathrm{Emb})\\ \mathrm{Output} & ={\mathrm{Norm}}_{4}({\mathrm{MLP}}_{2}{(\mathrm{Norm}}_{3}(\mathrm{Dim}\_\mathrm{Enc}))) \end{array}$$

### 4.3. Training Process of MultiScaleFormer Performance Degradation Predictor

**Figure 6:**
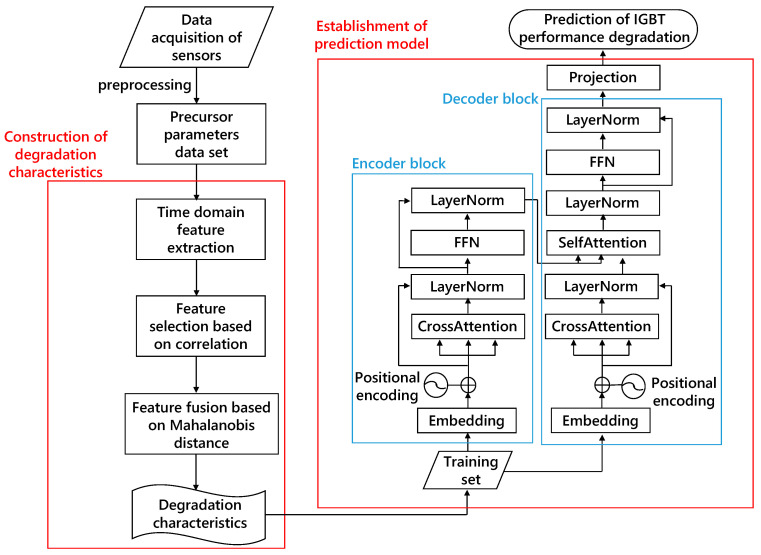
Flow chart of IGBT degradation prediction.

The experimental process and MultiScaleFormer structure for IGBT gate oxide performance degradation prediction are shown in Figure 6.

The left part of Figure 5 illustrates the feature engineering of IGBT gate aging data. Approximately 400,000 sampled matrices collected by sensors are preprocessed, resulting in 700 cycles of performance degradation metrics. Since IGBT performance degradation prediction is highly related to historical state data rather than the direct correspondence between time and state, the input sequence length is set to six and the prediction sequence length to one, meaning that the performance degradation metrics of six cycles are input into the prediction network each time, and the network provides the prediction for the next cycle.

The training process and detailed structure of the MultiScaleFormer are shown in the right part of Figure 5. The 700-cycle performance degradation metrics are divided into training, testing, and validation sets at a 7:2:1 ratio. The training set is used to fit the predictor, the validation set is used to check for overfitting or underfitting, and the testing set is used to test the accuracy of the predictor.

In this article, the mean squared error (MSE) loss function is used to calculate the loss between the predicted and true values, and the adaptive moment estimation (Adam) optimizer is used for model optimization. The initial learning rate is set to 8 × 10^−5^, and the model converges within 20 epochs. After training, the model and corresponding parameters are saved for testing set prediction.

### 4.4. IGBT Gate Oxide Performance Degradation Prediction Experiment

Using I_GES_ as the precursor parameter for gate oxide degradation, we construct performance degradation metrics for IGBT gate oxide and establish an IGBT performance degradation prediction model based on the MultiScaleFormer network. The data from the training set are used for training, and real-time predictions for the IGBT over 140 cycles are made. To verify the effectiveness of the MultiScaleFormer network, we conduct comparative experiments using Gaussian Prediction Regression (GPR), Support Vector Regression (SVR), LSTM, CNN, CNN_LSTM, and the Transformer network.

**Figure 7:**
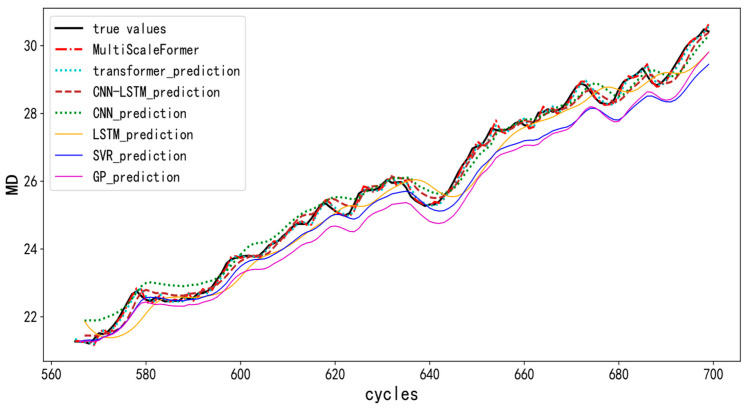
Forecast curves of MultiScaleFormer, Transformer, CNN-LSTM, CNN, LSTM, SVR, and GPR models.

In Figure 7, the horizontal axis represents the IGBT cycles and the vertical axis represents the IGBT performance degradation metrics (MD). A comparison between the various models’ predictions and true values (black solid line) is illustrated. The prediction curve of MultiScaleFormer (red dashed line) closely matches the true values, indicating a high prediction accuracy. Although the Transformer model (light blue dashed line) also performs well, it is slightly inferior to MultiScaleFormer. The CNN-LSTM model (brown dashed line) is stable, but deviates in some regions (e.g., cycles between 670 and 700). The CNN model (green dashed line) shows fluctuations, particularly with larger deviations in some regions. The LSTM model (orange solid line) performs well in some areas, but has a smoother overall prediction curve, failing to capture some detailed changes. The SVR (blue solid line) and GPR (purple solid line) prediction curves significantly deviate from the true values, performing poorly.

We quantitatively compare the prediction accuracies of different models using the mean absolute error (MAE) and record each model’s training time, as shown in Table 1.

From Table 1, it is evident that MultiScaleFormer exhibits the best prediction accuracy (lowest MAE of 0.0087). Despite its longer computation time, it is the optimal choice for tasks requiring a high precision. In contrast, the Transformer and CNN, despite having shorter computation times, offer a moderate prediction accuracy, making them suitable for tasks with lower precision requirements.

In summary, MultiScaleFormer is an efficient and high-precision prediction model. Its superior predictive performance can primarily be attributed to the following points: (i). Treating different time steps as separate tokens enhances the model’s ability to capture the relationships between time steps. (ii). The introduction of the cross-attention mechanism improves the feature representation richness and accuracy by integrating multi-source information. (iii). Multi-level information capture enhances the model’s generalization ability by capturing complex temporal and spatial relationships at different levels. These technical improvements enable MultiScaleFormer to excel in handling complex time series data, with a significantly better prediction accuracy and stability than other models.

## 5. Conclusions

In view of the degradation of IGBT gate oxide, this article takes the gate leakage current as a failure precursor parameter, analyzes it in the time domain, and obtains a performance degradation indicator (MD) that can characterize the IGBT gate oxide layer through feature selection, feature fusion, and noise reduction processing. In terms of the performance degradation prediction of the IGBT gate oxide layer, a prediction model based on the MultiScaleFormer network is constructed, and the effectiveness of the model is verified by using NASA’s public dataset. The main contributions of this paper are as follows.

1. The failure modes and precursor parameters of IGBTs were systematically sorted out, and the mechanism of gate oxide performance degradation and the engineering application method of using the gate leakage current to predict reliability were clarified and realized, including data preprocessing, feature selection, fusion, and filtering.
2. For the first time, the transformer network model was introduced for the analysis and prediction of the gate oxide performance degradation and failure precursors of IGBTs, and the usability of the transformer model in IGBT degradation prediction was verified.
3. A new prediction model which we call “MultiScaleFormer” was creatively proposed, which cleverly combined the links of cross-attention, showed an excellent prediction performance, and improved the error by an order of magnitude compared with the early CNN_LTSM, Transformer, and other models.

In summary, a MultiScaleFormer prediction model for the degradation of IGBT gate oxide performance was constructed in this article, the gate leakage current was selected as the failure precursor parameter, and the unified indicator MD for IGBT aging was obtained through correlation analysis and screening, the Mahalanobis distance fusion, and Kalman filtering. A variety of prediction models, including CNN, CNN-LSTM, SVR, GP, transformer, and Multiscaleformer, were used to test and compare. The performance of Multiscaleformer degradation prediction was the best, with a final average absolute error was as low as 0.0087, which indicated the feasibility of gate leakage current as a precursor parameter and the accuracy, reliability, and advancement of the Multiscaleformer prediction model.

## Figures and Tables

**Table 1:** Training times and MAEs of different prediction models.

Models	Time (s)	MAE
MultiScaleFormer	53	0.0087
Transformer	11	0.0827
CNN_LSTM	294	0.0370
LSTM	48	0.0409
CNN	16	0.0556
SVR	0.005	0.4561
GPR	0.14	0.5935

## Data Availability

The dataset used in this paper for supporting that our proposed framework is effective for aging prediction of IGBT grid oxide layer was provided by the NASA Prognostics Center of Excellence (PCoE). The data for download is available on https://data.nasa.gov/download/nk8v-ckry/application%2Fzip, accessed on 26 April 2024.
